# Development of an *Ex Vivo* Porcine Lung Model for Studying Growth, Virulence, and Signaling of Pseudomonas aeruginosa

**DOI:** 10.1128/IAI.01554-14

**Published:** 2014-08

**Authors:** Freya Harrison, Aneesha Muruli, Steven Higgins, Stephen P. Diggle

**Affiliations:** School of Life Sciences, Centre for Biomolecular Sciences, University of Nottingham, Nottingham, United Kingdom

## Abstract

Research into chronic infection by bacterial pathogens, such as Pseudomonas aeruginosa, uses various *in vitro* and live host models. While these have increased our understanding of pathogen growth, virulence, and evolution, each model has certain limitations. *In vitro* models cannot recapitulate the complex spatial structure of host organs, while experiments on live hosts are limited in terms of sample size and infection duration for ethical reasons; live mammal models also require specialized facilities which are costly to run. To address this, we have developed an *ex vivo* pig lung (EVPL) model for quantifying Pseudomonas aeruginosa growth, quorum sensing (QS), virulence factor production, and tissue damage in an environment that mimics a chronically infected cystic fibrosis (CF) lung. In a first test of our model, we show that *lasR* mutants, which do not respond to 3-oxo-C_12_-homoserine lactone (HSL)-mediated QS, exhibit reduced virulence factor production in EVPL. We also show that *lasR* mutants grow as well as or better than a corresponding wild-type strain in EVPL. *lasR* mutants frequently and repeatedly arise during chronic CF lung infection, but the evolutionary forces governing their appearance and spread are not clear. Our data are not consistent with the hypothesis that *lasR* mutants act as social “cheats” in the lung; rather, our results support the hypothesis that *lasR* mutants are more adapted to the lung environment. More generally, this model will facilitate improved studies of microbial disease, especially studies of how cells of the same and different species interact in polymicrobial infections in a spatially structured environment.

## INTRODUCTION

Pseudomonas aeruginosa commonly causes nosocomial infections and is a particular danger for people with cystic fibrosis (CF), in whom it establishes chronic lung infections (1). These are virtually impossible to clear with current therapeutic regimes, due to ciliary malfunction, the build-up of adhesive mucus in the CF airways (2), antibiotic resistance, and P. aeruginosa's ability to produce protective polysaccharide capsules (3). People with CF experience decades of chronic infection with repeated episodes of acute pulmonary exacerbation (1). During this time, P. aeruginosa evolves and diversifies; mutants with altered production of virulence factors are commonly isolated from patients (4–11), as are mutants that are impaired in quorum sensing (QS) (9, 10, 12).

Our understanding of the evolutionary pressures on P. aeruginosa during chronic lung infection and how these may be mediated by population or wider microbial community structure in these spatially structured organs is currently limited (13–15). Yet the evolutionary ecology of lung infections is likely a key factor in morbidity and response to clinical interventions (13, 16).

To clarify the role played by different P. aeruginosa virulence factors and by intermicrobial interactions in chronic infection, we need model systems that closely mimic a lung environment but are tractable in the lab and amenable to high-throughput experiments. A variety of *in vitro* growth conditions and insect or rodent hosts have been used to study P. aeruginosa populations. The pros and cons of these systems are outlined in Table 1, along with those of an underused model host: *ex vivo* sections of porcine lung. This model is useful for several reasons. First, pigs are arguably better models for studying human disease than are rodents or invertebrates (17–19). Second, lungs can be obtained from butchers: since little or no lung tissue is used in food production, lungs are (i) cheap and (ii) a waste product whose use does not raise ethical questions about the slaughter of animals for research. Third, many small sections of tissue can be kept in culture for several weeks (20, 21). Finally, and crucially, the spatial structure of the tissue is retained, and microbes can be visualized within the tissue by conventional or confocal microscopy. Histopathological changes can also be examined.

**TABLE 1:** Comparison of different model systems for studying pathogen social behavior and virulence

Category or concern	Data for model system (reference)				
	Petri dish/test tube	Live invertebrate	Live mammal	Human cell or tissue culture	*Ex vivo* pig lung culture
Example studies	References 22–26	Waxworm (27, 28), fruit fly (16), or nematode (29, 30)	Usually mouse (14, 31–36), occasionally other small mammals (37); also, a CF pig model exists (38)	References 39–41	References 20, 21, 42
Chemical environment	Can be controlled to mimic CF sputum (24, 25)	Not known	Mouse metabolome (17) and gene expression (19) very different from human	Controllable and can be made to mimic *in vivo* conditions	Metabolome more similar to human than a mouse is (17)
Spatial structure	Can be controlled, but artificial	Limited	Burn wounds, limited; lung infections, yes	Possible with scaffolding or organ sections	Very similar to human lung (18)
Immune system	None	Limited similarity to humans	Limited similarity to humans	Human	Very similar to human (18) but largely lost *ex vivo*
Infection time scale	Can study hundreds to thousands of generations	Acute: host dies very quickly	At best semichronic; rodent lung infections tend to be acute (days), though can sometimes last 1–4 weeks (43, 44); wound infections are usually limited to ca. 3 weeks (K. Rumbaugh, pers. commun.)^*a*^	Days to weeks	Not known
Large sample sizes (tens plus) possible?	Yes	Yes	No, due to cost and ethical considerations	Not usually	Yes
Cost	Low-medium	Low	High	Medium to set up, low to run	Low
Ease of method	Simple, requires only general microbiology techniques	Must learn how to inoculate but otherwise simple	Requires specialized expertise, an animal license and often a dedicated animal worker to carry out inoculation	Requires expertise and dedicated lab space/equipment to minimize risk of contaminating cell lines	Lungs are readily obtained from commercial butchers; we developed dissection, infection, and culture techniques in ca. 3 months
Ethical considerations	None	None	Yes—and limit sample size/infection duration	Minimal (donor informed consent must be obtained)	None if obtained from animals slaughtered for meat; little or no tissue is used for human consumption, so lungs are basically a waste product.
Review articles		References 45, 46	References 37, 46, 47	References 46, 48	

a Infection duration depends on local rules governing animal welfare; e.g., in the United Kingdom, animals must be euthanized when the symptoms of infection become too severe. pers. commun., personal communication.

We developed this model for quantitative studies of P. aeruginosa growth and exoproduct production. We focused on the well-characterized PAO1 wild-type (WT) strain and two *lasR* mutant strains which do not respond to the QS signal *N*-(3-oxododecanoyl)-l-homoserine lactone (3-oxo-C_12_-HSL) (49). QS controls the expression of various exoproducts (49) and facilitates the establishment of acute infection (31–36). However, mutants that have lost LasR function and so do not respond to 3-oxo-C_12_-HSL commonly arise in chronic CF infections (9, 10, 12) and ventilator-associated pneumonia (50). There is debate over whether *lasR* mutants are social “cheats” that benefit from the presence of WT cells (51, 52) or whether they are adapted to the chronic lung environment. Resolving this question is important because it will affect the likely clinical success of QS inhibitors, which have been suggested as novel antivirulence agents and antibiotic adjuvants (53, 54). We therefore compared the growth of *lasR* mutants with that of the WT in single-genotype and mixed infections in *ex vivo* pig lungs (EVPL). We also measured the production of 3-oxo-C_12_-HSL and of two groups of virulence factors whose expression is regulated by QS and that are linked with virulence in acute infection or with acute exacerbation and declining lung function in people with CF: tissue-degrading proteases (55) and redox-active phenazines (55–57). We also assayed for the siderophores pyoverdine and pyochelin, since these have been shown to be necessary for acute infection (27, 58) and their role in chronic infection has been much discussed (59–62).

We report three key results: (i) we can detect differential production of 3-oxo-C_12_-HSL, protease, and phenazine compounds by WT and QS mutant P. aeruginosa colonizing EVPL; (ii) consistent with this, *lasR* mutants cause less pathological change to the host tissue; and (iii) *lasR* mutants grow as well as or better than the WT in EVPL in single infections, and a marked *lasR* mutant had fitness equal to that of the WT in a mixed infection. Therefore, in this context, *lasR* mutants do not behave as social cheats: rather, they grow well in this chronic infection model.

## MATERIALS AND METHODS

### Bacterial strains and culture conditions.

The Nottingham PAO1 strain of P. aeruginosa was used as the wild type (WT). A PAO1 mutant, carrying a gentamicin resistance cassette inserted into the *lasR* gene, was used as a marked *lasR*-null mutant (*lasR*::Gm) (26). For comparison, an unmarked PAO1 clone with a clean deletion of *lasR* (Δ*lasR*) was also used. Preliminary work suggested that levels of phenazines produced in our infection model were too low to be assayed via spectrophotometry, so we used PAO1 WT and PAO1Δ*lasR* strains carrying a reporter construct for one of the main phenazine biosynthetic operons (*phzA1-luxCDABE* fusion; S. Higgins, S. Heeb, G. Rampioni, P. Williams, N. Krasnogor, and M. Cámara, unpublished data). Infected cubes of lung tissue were cultured in artificial sputum medium (ASM) (24) for 24 h at 37°C on an orbital shaker. ASM mimics the chemical composition of CF sputum but is not viscous. All media used were supplemented with 50 μg/ml ampicillin to minimize the growth of any resident bacteria present in the lung cubes.

### Preliminary work and observations.

Lungs were purchased from a butcher (A Holmes and Son, Coalville, Leicestershire, United Kingdom). We conducted preliminary work on five lungs and determined that the tissue was healthy and not damaged by the process of dissecting and preparing tissue (see Fig. S1 in the supplemental material). We used the work of Nunes et al. (42) as a starting point to develop a protocol for dissecting out relatively regular cubes of alveolar tissue of approximately 5 mm^3^ (125 μl), inoculating with ca. 10^4^ to 10^5^ P. aeruginosa cells, and culturing the infected cubes in ASM for up to 7 days. Finally, we determined that we could visualize luminescent reporter bacteria in the cubes, homogenize infected tissue to recover live bacteria, and conduct quantitative assays with lung homogenate for the presence of 3-oxo-C_12_ HSL, total protease, pyocyanin, and light production by luminescent (*lux*) reporters. We also verified that resident lung bacteria were present at very low levels, being almost entirely outcompeted by P. aeruginosa in infected tissue.

### Ethics statement.

All lung material was purchased from a retail butcher and was sourced from animals already slaughtered for meat; ethical approval was therefore not required for this study.

### Preparation of lung material.

**FIG 1:**
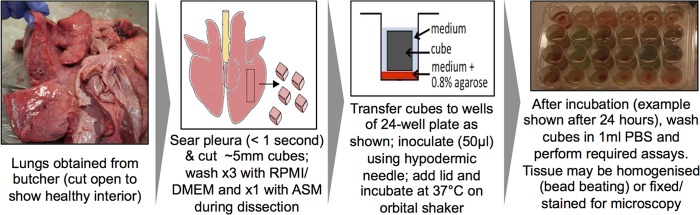
Schematic of the final protocol for preparation, infection, and culture of EVPL.

The final protocol for the preparation, inoculation, and culture of EVPL is shown in Fig. 1. Cubes of approximately 5 mm^3^ were dissected from the ventral surface of the left caudal lobe of three sets of lungs using a sterile mounted razor blade. Large bronchioles and veins were avoided in order to keep the cubes as comparable as possible. Prior to dissection, the ventral surface of the pleura was briefly (<1 s) seared with a hot pallet knife to kill surface contaminants from the abbatoir or butcher's shop. This also rendered the pleura easier to cut. During dissection, the tissue to be used was washed three times with cell culture medium (1:1 mix of RPMI 1640 and Dulbecco's modified Eagle medium [DMEM]; Sigma-Aldrich). The cubes were then washed for a fourth time in ASM. Preliminary work confirmed that searing and washing did not cause any visible damage to the pleura (light microscopy of formalin-fixed tissue) and that these processes reduced the numbers of contaminating and/or resident bacterial cells present in the cubes. We aliquoted 400 μl ASM supplemented with 0.8% agarose to individual wells of a sterile 24-well plate (to provide a soft surface for the tissue to sit on) and placed cubes singly in wells on this surface. Cubes were covered with 500 μl liquid ASM. As a control experiment to explore the growth of the bacterial strains in the absence of lung tissue, cultures were set up exactly as described above, but in place of the lung cube, an extra 125 μl liquid ASM was added. Three experimental replicates of this experiment were performed; in each case, five populations each of WT, *lasR*::Gm, Δ*lasR*, and WT plus *lasR*::Gm bacteria in a 1:1 mix were inoculated.

### Inoculation of lung tissue.

Bacterial strains were grown overnight in lysogeny broth (LB), washed twice in phosphate-buffered saline (PBS), and resuspended in ASM. Cubes were inoculated with ca. 10^4^ washed overnight-culture cells in 50 μl ASM—or as a mock-infection control, with 50 μl sterile ASM—using a 30-gauge needle attached to a disposable 1-ml syringe. Cubes were then incubated for 24 h at 37°C on an orbital shaker. Figure S2 in the supplemental material shows a schematic of the experiment.

### Assays.

After incubation, cubes were rinsed in 1 ml PBS to remove loosely adhering cells. Growth of bacteria was assayed by homogenizing cubes individually in 500 μl phosphate-buffered saline with metal bead tubes (Cambio) using a Precellys24 homogenizer, serially diluting the homogenate and plating aliquots on LB plates to obtain single colonies. To score the relative frequencies of WT and *lasR* mutant cells in mixed infections, aliquots were replica plated onto LB plus 20 μg/ml gentamicin. In mixed infections, the relative fitness of the mutant was calculated as follows: *v* = [*x*_2_(1 − *x*_1_)]/[*x*_1_(1 − *x*_2_)], where *x*_1_ and *x*_2_ are the initial and final frequencies of the mutant in the population, respectively. When the two genotypes have equal fitness, *x*_1_ = *x*_2_ and *v* = 1. *v* values of <1 reflect the mutant being outcompeted by the WT, and values of >1 indicate that the mutant outcompetes the WT. To quantify QS signals, total protease, pyocyanin, and the siderophores pyoverdin and pyochelin, an aliquot of the homogenate was diluted 10-fold in PBS and filtered using a 0.2-μm syringe-driven filter unit to remove cells. This was stored at −20°C. The amount of the QS signal 3-oxo-C_12_-HSL in the diluted homogenates was quantified using the pSB1075 Escherichia coli biosensor (63); briefly, 100 μl of each homogenate was mixed with 100 μl of an overnight biosensor culture diluted to an optical density at 600 nm (OD_600_) of ∼0.1, and the luminescence/OD_600_ for each culture measured after 30 min of incubation at 37°C in a 96-well plate. The assay was calibrated using purified 3-oxo-C_12_-HSL. To measure total protease, 100 μl homogenate was mixed with 5 mg azocasein dissolved in 900 μl 100 mM Tris-HCl plus 1 mM CaCl_2_, and the mixture was incubated with shaking for 15 min at 37°C; the reaction was then stopped by adding 500 μl 10% trichloroacetic acid, and the absorbance of the supernatant was read at 400 nm. This assay was calibrated using known concentrations of purified proteinase K. Pyocyanin was quantified by measuring absorbance of homogenates at 695 nm, pyoverdine by exciting with light at 400 nm and measuring fluorescence at 460 nm (64) and pyochelin by exciting at 350 nm and measuring fluorescence at 430 nm (65). To assay activity of the *phzA1* phenazine operon by reporter bacteria, aliquots of nonfiltered, undiluted homogenate were assayed for luminescence. Spectrophotometric assays were carried out using either a Tecan Infinite F200 Pro instrument (3-oxo-C_12_-HSL biosensor, protease, luminescence) or a Molecular Devices SpectraMax M2 instrument (pyoverdine, pyochelin, and pyocycanin). Finally, to assess tissue damage and bacterial growth, cubes were fixed in formalin, sectioned, and stained with hematoxylin and eosin (H&E) and Gram's stain and inspected under a light microscope (Nikon Eclipse 50i with Digital Sight DS-U3 camera).

### Statistical analysis.

Analysis of variance (ANOVA) with type II sums of squares (*car* package [66] in R 2.14.0 [67]) was used to test for main effects of lung and inoculum (sterile ASM and/or the different bacterial strains) and for differential effect of inoculum in different lungs (lung-inoculum interaction when the sterile ASM control was included in the analysis, lung-strain interaction when the sterile ASM control was excluded) on dependent variables. Data on total number of CFU, relative fitness of the *lasR*::Gm mutant, 3-oxo-C_12_-HSL concentration, protease concentration, and phenazine reporter expression were transformed using the natural logarithm when they were used as dependent variables, in order to meet the assumptions of ANOVA. All *P* values are given for two-tailed tests.

## RESULTS

### P. aeruginosa causes visible tissue damage.

As shown in Figure S1 in the supplemental material, fresh uninfected lung cubes appeared healthy, with open alveoli surrounded by thin, well-defined epithelium. To explore the effects of *lasR*-mediated QS on growth and virulence, cubes were inoculated with ca. 10^4^ washed overnight-culture cells of WT PAO1, two independent *lasR* mutants (the insertional mutant PAO1 *lasR*::Gm and the clean knockout PAO1 Δ*lasR*), a mix of PAO1 and PAO1 *lasR*::Gm, or a phenazine bioreporter strain constructed in a WT or Δ*lasR* background. As a mock infection control, cubes were inoculated with 50 μl sterile artificial sputum medium (ASM). A visual overview of the final dissection and infection protocol is given in Fig. 1, and a schematic of the experimental design is given in Fig. S2.

**FIG 2:**
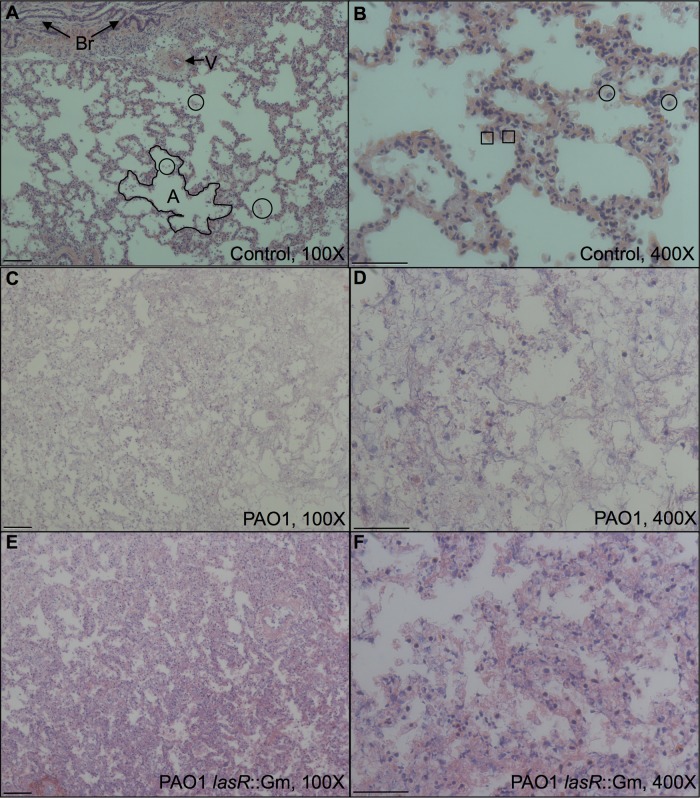
Micrographs of tissue after 24 h in ASM, fixed and stained with H&E, which colors nuclei dark blue and other structures (cytoplasm, collagen, etc.) pink. (A and B) Mock-infected control; (C and D) infected with WT P. aeruginosa; (E and F) infected with the *lasR*::Gm mutant. Panels A, C, and E show tissue at magnification ×100 with a 100 μM scale bar; panels B, D, and F show tissue at magnification ×400 with a 50 μM scale bar. In panel A, note two bronchioles (Br) with diagnostic folded epithelium of brush border, example of a blood vessel (V), and lace-like pattern of alveoli defined by thin epithelium (example outlined; A). Small patches of cellular debris are visible in the alveoli (three examples are circled). In panel B, occasional cells with horseshoe-shaped nuclei (circled) are visible, which may represent neutrophils, along with enucleate red blood cells (two examples are boxed). Note in panels C and D the loss of clear epithelium, lower number of nuclei, and a decreased volume of airspace. In panels E and F, this change is less extreme, with thickened outlines of epithelium still discernible.

Mock-infected cubes retained their gross structural integrity over 24 h at 37°C, while infected cubes lost their shape and became soft, with visible green P. aeruginosa growth (see Fig. S3 in the supplemental material). Microscopy of fixed and stained tissue sections showed that mock-infected lungs showed minimal histopathological changes compared with lung tissue that was fixed and sectioned prior to infection, with only small amounts of apoptotic/necrotic debris in the alveoli (Fig. 2A and B); in most cases, Gram staining did not show the presence of resident bacteria. Some sections of mock-infected tissue exhibited areas of reduced alveolar volume reminiscent of areas of inflammation in living tissue, and the least histologically normal sample also contained large numbers of Gram-negative rods; it is highly unlikely that these were P. aeruginosa, since we never recovered P. aeruginosa when we plated out mock-infected lung homogenate. As exemplified in Fig. 2C and D, sections of tissue infected with PAO1 WT had no remaining alveolar structure, and far fewer cell nuclei were visible than in mock-infected tissue. Tissue preservation appeared slightly better in lung tissues infected with the *lasR*::Gm mutant; as exemplified in Fig. 2E and F, these were more reminiscent of highly inflamed tissue. In infected tissues, Gram staining revealed large numbers of Gram-negative rods, which we presume to be P. aeruginosa (see Fig. S4 in the supplemental material).

### *lasR* mutants do not show a growth disadvantage in EVPL.

**FIG 3:**
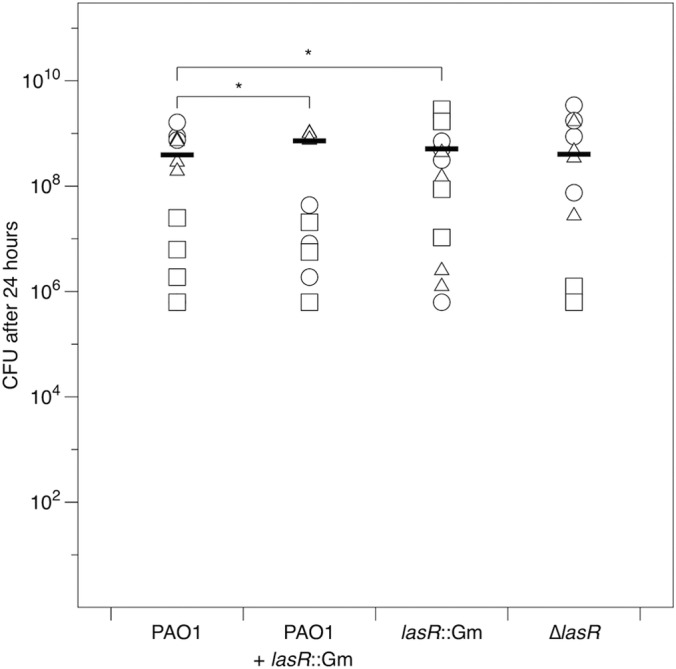
Number of CFU of P. aeruginosa recovered from *ex vivo* pig lung cubes after 24 h of incubation in artificial sputum medium. Different symbols show cubes from independent lungs, and bars denote overall means. Where pairwise differences between strains were found to be significant (*P* < 0.05) using Tukey HSD tests, this is indicated with an asterisk.

We compared the fitness of WT and *lasR* mutant genotypes in single and mixed infections of EVPL. P. aeruginosa grew in the lungs (Fig. 3), reaching final densities of 6 × 10^5^ to 4 × 10^9^ CFU per cube (5 × 10^3^ to 3 × 10^7^ CFU per mm^3^ of tissue). The final density differed between strains (*F*_3,36_ = 4.48; *P* = 0.009) and between lungs (*F*_2,36_ = 19.2; *P* < 0.001); crucially, the different strains showed consistent differences in growth across the different lungs (interaction *F*_6,36_ = 0.830; *P* = 0.555). *Post hoc* Tukey honestly significant difference (HSD) comparisons showed that QS mutants grew as well (for the Δ*lasR* mutant, *P* = 0.297) or better (for the *lasR*::Gm mutant, *P* = 0.049) than the WT in single infections. The mixed infections were initiated with a mixture comprising ca. 60% WT/40% *lasR*::Gm mutant, and these frequencies did not change over the incubation period: the relative fitness of the mutant did not vary between lungs (ANOVA for effect of lung; *F*_2,7_ = 0.04; *P* = 0.97) and was not significantly different from 1 (*post hoc t* test, *t* = 0.62; *P* = 0.56).

To determine whether the relative fitness of the *lasR* mutant was due to growth in lung tissue and not simply to growth in ASM, we performed a control experiment in which the cube of EVPL was replaced with a corresponding volume of ASM. Pure cultures of the *lasR*::Gm and Δ*lasR* mutants grew to approximately half the density of the WT (*P* < 0.001 and *P* = 0.013, respectively); the final density of the mixed WT plus *lasR*::Gm population did not differ significantly from that of the WT (*P* = 0.060). These results are shown in Figure S5 in the supplemental material; *P* values are from *post hoc* tests after a fully factorial ANOVA testing for the effects of strain and experimental replicate (strain *F*_3,47_ = 7.69; *P* < 0.001). The *lasR*::Gm mutant was not able to take advantage of the WT in mixed culture; its relative fitness did not differ in pure and mixed culture (fully factorial ANOVA including experimental replicate: strain *F*_1,23_ = 3.40; *P* = 0.078) and was significantly <1 in both cases (*post hoc t* test, *t* = 2.25; *P* = 0.034). This appeared to be due to the WT growing better in the absence of EVPL than in its presence, but further work is needed to explore this.

### P. aeruginosa virulence factor expression in EVPL is QS dependent.

**FIG 4:**
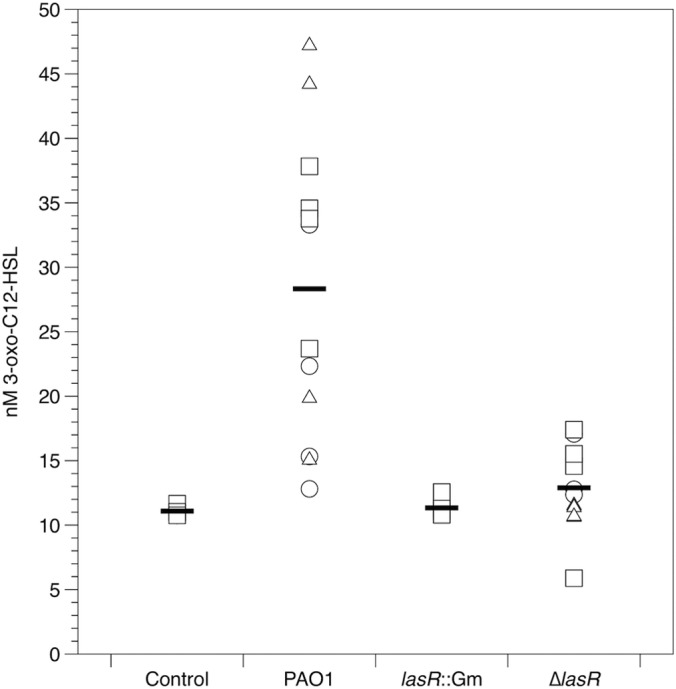
3-oxo-C_12_-HSL signal in mock-inoculated and P. aeruginosa-infected lung cubes after 24 h incubation. Different symbols show cubes from independent lungs, and bars denote overall means. The amount of signal in the WT-infected cubes was significantly greater than that in cubes infected with the other three strains (Tukey HSD tests, *P* < 0.001).

We used an E. coli bioreporter (63) to measure 3-oxo-C_12_-HSL in cell-free homogenates of mock-infected and infected lung. As shown in Fig. 4, the amount of signal produced differed between inocula (*F*_2,36_ = 28.6; *P* < 0.001) but not between lungs (main effect, *F*_3, 36_ = 0.50; *P* = 0.611; interaction, *F*_6,36_ = 1.39; *P* = 0.24). This was due to the WT infection producing more signal than the control or *lasR* mutant infections (Tukey HSD tests, *P* < 0.001; mutant infections did not differ from the control [*P* > 0.7]). The WT produced, on average, 16 nM 3-oxo-C_12_ HSL (range, 6 to 47 nM). We reran these analyses excluding the mock-infected control and included total CFU in the cube as a covariate to eliminate the possibility that any differences between strains were due to variability in population density; the results were unchanged. Many secreted molecules that have been linked with virulence in acute infection models or with acute exacerbations or more rapid decline of lung function in CF are under QS control. These include tissue-degrading proteases (55) and redox-active phenazine compounds (55–57). We then sought to determine whether mutations in *lasR* and concomitant loss of 3-oxo-C_12_-HSL led to decreased production of protease, phenazines, and siderophores in EVPL.

**FIG 5:**
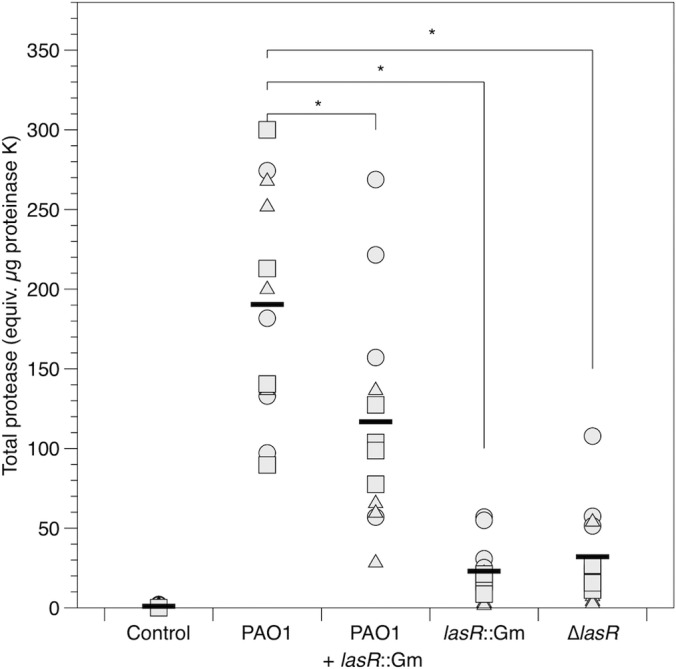
Total protease in mock-inoculated and P. aeruginosa-infected lung cubes after 24 h of incubation. Different symbols show cubes from independent lungs, and bars denote overall means. Where pairwise differences between strains were found to be significant (*P* < 0.005) using Tukey HSD tests, this is indicated with an asterisk.

Consistent with lower levels of tissue damage (Fig. 2), the *lasR* mutants produced less protease *per capita* than the WT (strain, *F*_3,36_ = 8.77; *P* < 0.001; lung, *F*_2,36_ = 12.5; *P* < 0.001; interaction, *F*_6,36_ = 1.28; *P* = 0.29), and this translated into much lower total protease in lung cubes (Fig. 5). Analysis including mock-infected cubes: inoculum, *F*_4,45_ = 148; *P* < 0.001; lung, *F*_2,45_ = 14.0; *P* < 0.001; interaction, *F*_8,45_ = 7.4; *P* < 0.001). On average, the total protease activities in cubes infected with the Δ*lasR* mutant and the *lasR*::Gm mutant were 16% and 12%, respectively, of that measured in WT-infected cubes. Mock-infected cubes contained no measurable protease (*t* = 0.581; *P* = 0.56), underlining the loss of immune activity in this model. We reran the *per capita* and total protease analyses excluding the mock-infected control and included total CFU in the cube as a covariate to eliminate the possibility that our results were due to variability in population density; the results for the main effects of lung and strain were unchanged, but the lung-strain interaction became nonsignificant in both cases (*P* > 0.1).

**FIG 6:**
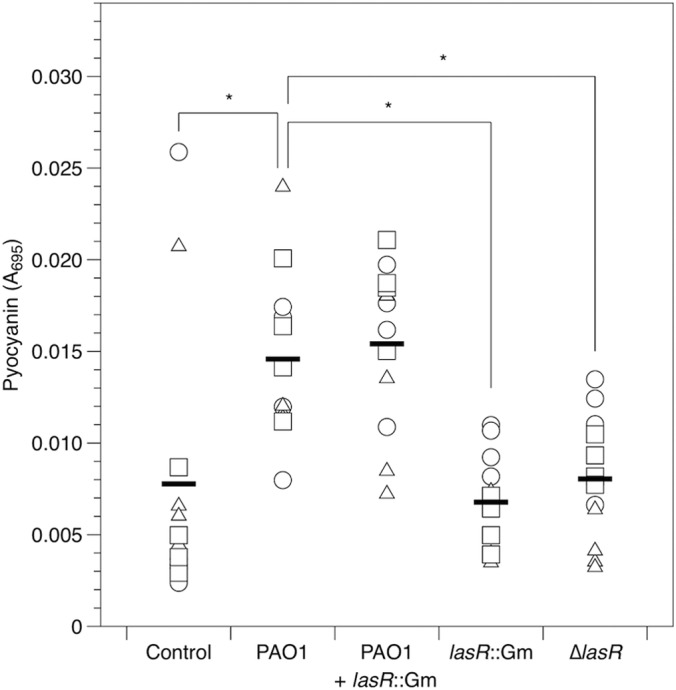
Pyocyanin (*A*_695_) in mock-inoculated and P. aeruginosa-infected lung cubes after 24 h of incubation. Different symbols show cubes from independent lungs, and bars denote overall means. Where pairwise differences between strains were found to be significant (*P* ≤ 0.006) using Tukey HSD tests, this is indicated with an asterisk.

**FIG 7:**
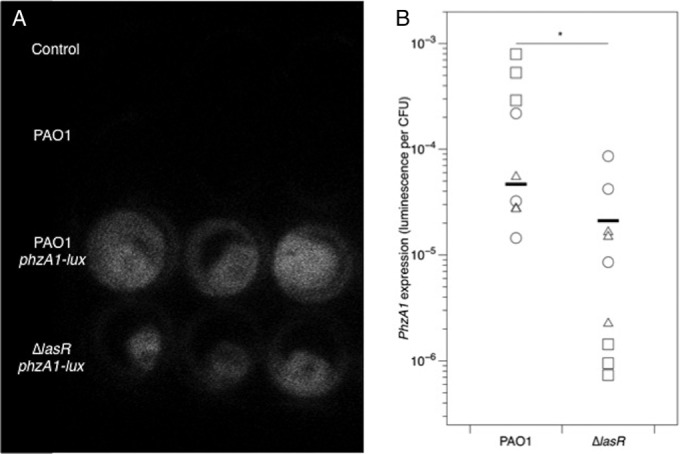
(A) Photon-counting image of cubes taken from one lung after 24 h of incubation. Mock-inoculated cubes and cubes infected with unlabeled NPAO1 show no luminescence, and cubes infected with *phzA1-luxCDABE* reporters show luminescence. (B) Per-CFU expression of *phzA1* by P. aeruginosa in lung cubes (arbitrary luminescence units divided by CFU and blanked on samples from cubes infected with the unlabeled NPAO1). Different symbols show cubes from independent lungs, and bars denote overall means. The asterisk denotes a significant difference between strains in ANOVA (*P* < 0.001).

Similar results were obtained for the phenazine compound pyocyanin (Fig. 6. Analysis including mock-infected cubes: inoculum, *F*_4,45_ = 12.7; *P* < 0.001; lung, *F*_2,45_ = 2.47; *P* = 0.096; interaction, *F*_8,36_ = 2.18; *P* = 0.047; dropping two outliers from the control group did not affect these results). Visual inspection of cubes infected with a luminescent reporter for the phenazine biosynthetic operon *phzA1* using a photon-counting camera confirmed that this operon was expressed in infected cubes (Fig. 7A), and plating confirmed that phenazine reporter constructs grew to similar densities regardless of whether they were in a WT or Δ*lasR* genetic background. A quantitative assay showed that per-CFU expression of luminescence by the *phzA1* reporter construct was lower in the Δ*lasR* background than in the WT (Fig. 7B, *F*_1,12_ = 37.9; *P* < 0.001); on average, expression in the Δ*lasR* background was 45% of that in the WT background, but the magnitude of this difference differed between lungs (main effect, *F*_2,12_ = 1.22; *P* = 0.33; interaction, *F*_2,12_ = 16.9; *P* < 0.001). Again, including total CFU in the cubes as a covariate did not affect the results for pyocyanin and *phzA1* reporter expression. We could not detect the primary and secondary siderophores pyoverdine and pyochelin in lung homogenates using excitation/emission assays, which have been shown to be sensitive to ≥10 μM pyoverdine (F. Harrison, unpublished data) and ≥2 μM pyochelin (65).

## DISCUSSION

### Tractability and potential of EVPL as an infection model.

*Ex vivo* sections of pig lung are a tractable model for studying P. aeruginosa growth and virulence. Mock-infected tissue was relatively histologically normal after 24 h of incubation in ASM at 37°C, and preliminary observations suggest that little further histological change occurs in mock-infected tissue after a further 6 days of incubation. P. aeruginosa cells could be visualized in EVPL using a light microscope and readily recovered from tissue. Cell-free suspensions of homogenized tissue could be assayed for a range of bacterial virulence factors. The relative growth of WT and *lasR* mutant bacteria in EVPL contrasted with the situation in ASM alone: in this setting, the WT outgrew the mutants by a factor of approximately 2:1, whereas in EVPL the mutants grew as well as or slightly better than the WT.

A key advantage of EVPL is the chance to study bacterial virulence factor production, growth, and cell-cell interactions in a spatially structured environment. A diverse literature has explored the potential effects on bacterial gene expression, growth, and virulence of interactions between cells of the same or different species (4, 16, 27, 68–73) and how population structure can affect these interactions (22, 23, 35, 74). However, while it is clear that the CF infection community is spatially structured on a gross anatomical level (15, 75, 76), we do not know whether this community is spatially ordered at a scale relevant to bacterial cell-cell interactions. This means that it is hard to assess the likely efficacy of proposed clinical interventions that rely on disrupting cell-cell interactions, such as QS inhibitors (54). Further, spatial structuring of bacterial populations will affect other processes relevant to the development of chronic infection, such as the dynamics of bacteriocin-producing and -sensitive strains (77), plasmid transfer (78), and the evolution of antibiotic resistance (79, 80). The potential to manipulate the infection community inoculated into EVPL, to study its evolution using conventional and confocal microscopy of sections taken at various times postinoculation, and to correlate aspects of community diversity and structure with histopathology and levels of virulence factors represents a significant opportunity to study the extent and consequences of cell-cell interactions in lung tissue.

Clearly, EVPL also has limitations, and future work must identify these, circumvent them where possible, and clarify the extent to which EVPL represents a chronically infected human lung. First, we must acknowledge the high variance in the data presented in this article. Since we had no *a priori* expectations of the likely level of variability or ease of replication, this should be viewed mainly as a proof-of-principle study which can be built upon by ourselves and others. As a result of this work, we now know that we can readily access and process lungs in batches and easily cut several dozen regular cubes of tissue from each lung. This knowledge, along with the ability to use the data presented here in power calculations, will allow researchers to design larger-scale experiments which provide more reliable estimates of between-strain or between-genotype differences. Second, if we are to determine how well this model recapitulates chronic infection in humans, a detailed exploration of the chemical environment in EVPL and how this coevolves with infecting microbes over time (days to weeks) is required. For instance, we do not yet know whether the reported chemical similarities between human and pig lung are maintained in this *ex vivo* system or whether the ASM needs to be modified when it is used in conjunction with tissue (for example, ASM contains iron, but if this is also supplied in abundance by the lung tissue, then overall levels of bioavailable iron may be unrealistically high, and this could explain why siderophore gene expression appeared to be switched off in our experiment). Further, the oxygen regime in infected lungs is likely to be an important factor, and infection foci may become less aerobic over the course of infection (81, 82). Future work could address how and when oxygen levels change inside sections of EVPL and how this affects the growth and virulence of P. aeruginosa. Finally, a careful comparison of results obtained in EVPL with those obtained from live animal models, with clinical data from CF patients where applicable, will help us to determine whether the differences we observe between genotypes in this model are likely to be meaningful *in vivo*.

### Role of QS and fitness consequences of *lasR* mutation in EVPL.

3-Oxo-C_12_-HSL accumulated to nanomolar levels after 24 h of infection with WT PAO1. It is hard to know how this corresponds to the level of expression in CF lungs, since studies using various assay methods report concentrations ranging from femtomolar to micromolar in CF secretions and tissues (83–88). We detected significant differences between WT PAO1 and *lasR* mutants, which do not respond to 3-oxo-C_12_-HSL. *lasR* mutants produced no detectable 3-oxo-C_12_-HSL and significantly less protease and pyocyanin than the WT; further, expression of one of the phenazine biosynthetic operons, *phzA1*, was significantly reduced. Consistent with these results, *lasR* mutant-infected tissue exhibited qualitatively less tissue damage. We therefore conclude that P. aeruginosa “senses a quorum” in EVPL. Moreover, our results are consistent with reports that P. aeruginosa isolates from CF patients undergoing periods of acute exacerbation overproduce various QS-dependent exoproducts (8, 55, 89) and that in P. aeruginosa mouse infection models, areas of tissue with higher *N*-acylhomoserine lactone (AHL) concentrations exhibit more severe pathological changes (86), and *lasR* mutants cause less tissue damage than the WT (36). That our *lasR* mutants showed reduced pyocyanin production is interesting, because in standard laboratory medium *in vitro*, *lasR* mutants have been reported to produce significantly more pyocyanin than the WT (90, 91). In contrast, other studies have shown that among P. aeruginosa clones isolated from CF patients, *lasR* mutation is often associated with a loss of pyocyanin production *in vitro* (55; see also reference 92).

A key finding from our study is that *lasR* mutants grew as well as or better than the WT in EVPL. This is noteworthy because there has been considerable debate about the evolutionary dynamics of *lasR* mutants in chronic infection. There are at least three possible explanations for the presence of *lasR* QS-blind mutants in chronic P. aeruginosa infections. First, loss of QS response could be adaptive, conferring a growth or persistence advantage in the context of an established infection. Second, *lasR* mutants may act as social “cheats” and persist because they take advantage of coinfecting QS-proficient genotypes, whose QS-dependent exoproducts may benefit any cell in the vicinity, regardless of its own level of production (22, 93–96). Third, QS-blind mutants may be maladaptive but arise due to recurrent mutation and persist at low frequencies due to stochastic evolutionary drift. It is difficult to choose which of these alternatives (if any) is correct, because there is very little quantitative data on the frequency of QS-blind mutants within chronically infected hosts and how this changes (or not) over time.

Generally, the first hypothesis, that QS-blind mutants have a fitness advantage, has had little support, because loss of *lasR* function reduces the ability of P. aeruginosa to establish acute infections (31–36). We also found that in ASM in the absence of pig lung, *lasR* mutants were less fit than the WT. These observations, combined with demonstrations that *lasR* mutants can act as WT-exploiting cheats in some situations *in vitro* (22, 26, 97, 98) and in acute burn wound infections (32, 35), have led some researchers to give the second hypothesis serious consideration. This has led to the idea of deliberately introducing cheating mutants to trigger population collapse or to act as “Trojan horses” for carrying useful alleles (e.g., antibiotic susceptibility) into infectious populations (e.g. see reference 99).

Our result is not consistent with the “social cheat” hypothesis. Rather, it adds weight to the first hypothesis: that loss of LasR function enhances growth in chronic infection. The chemical environment in chronically infected, damaged tissues may confer a growth advantage on *lasR*-null mutants; D'Argenio et al. (100) report that the relative growth of WT and *lasR*-null monocultures *in vitro* is dependent on medium composition and that in some media, *lasR*-null mutants outgrow the WT. In addition, Duan and Surette (101) show that changes to medium composition can change the way that the QS system reacts to cell density. Moreover, in one of the few studies to track the frequency of *lasR*-null P. aeruginosa in human patients over the course of infection, Köhler et al. (50) interpret their data as showing that *lasR* mutants are cheats, but they report that patients colonized only by *lasR* mutants had bacterial loads similar to those of patients colonized only by the WT, and this strongly suggests that these mutants are not impaired in chronic persistence. For a detailed discussion of the evidence for adaptive loss of LasR, we refer the reader to the review by Heurlier et al. (102), and for a detailed discussion of social cheating, see Ghoul et al. (51).

We could not detect production of the siderophores pyoverdine and pyochelin in EVPL. While siderophores are necessary for acute infections of mice (58) and waxmoth larvae (27), their role in chronic CF lung infection is unclear because tissue damage and low oxygen levels may render iron more accessible (103–106). Studies of CF sputum samples have shown that siderophores are sometimes, but not always, present at detectable levels (61, 62).

### Future directions.

Further optimization of EVPL could render it a realistic, ethical, and high-throughput model for studying the evolutionary ecology and pathology of chronic lung infection. As discussed above, several questions regarding the biological realism of the model remain to be answered, but the methodology we have developed for processing and handling lung tissue will allow us and others to address these in detail. The model as presented here produced more extensive and less localized tissue damage than seen in postmortem CF lungs, but since we inoculated with a high dose of bacteria and used aerobic culture conditions, this is not surprising. Now that we have demonstrated the potential of EVPL, the next steps in developing the model will be to find conditions that produce stable and long-lived infections (e.g., by titrating the number of cells inoculated, culturing under microaerobic or anaerobic conditions, and simulating key aspects of the host immune response). In this study, we used alveolar tissue to minimize between-cube variation in structure, but future work could focus on dissecting regular sections of bronchiole, since these are the foci of infection in human CF lungs (107). A comparison between lung cubes obtained from healthy pigs and lung cubes taken from pigs genetically engineered to express human CF mutations (114) may also help to validate the system. In the future, EVPL could especially enhance research into interactions between microbes in multispecies infections, which are the norm in CF (13, 15, 108–110) and are increasingly recognized as important in other respiratory diseases, such as chronic obstructive pulmonary disease (COPD) and asthma (111–113).

## Supplementary Material

Supplemental material
