# Supplementary material for: Development of an Ex Vivo Porcine Lung Model for Studying Growth, Virulence, and Signaling of Pseudomonas aeruginosa
Source: Infect Immun. 2014 Aug;82(8):3312–23. doi: 10.1128/IAI.01554-14 (PMC4136229; doi:10.1128/IAI.01554-14)
Supplement: Supplemental material [file supp_82_8_3312__index.html]

Development of an Ex Vivo Porcine Lung Model for Studying Growth, Virulence, and Signaling of Pseudomonas aeruginosa — Supplemental material 

# Development of an *Ex Vivo* Porcine Lung Model for Studying Growth, Virulence, and Signaling of Pseudomonas aeruginosa

## Supplemental material

**Files in this Data Supplement:**

- Supplemental file 1 -

  Fig. S1. Micrograph of lung tissue prior to preparation. Fig. S2. Schematic of the experiment, showing numbers of cubes dissected from lungs and assigned to different treatments/assays. Fig. S3. Example of lung cubes after 24 hours of incubation at 37°C in ASM plus washing in PBS. Fig. S4. Gram-stained sections of tissue infected with WT PAO1 (a) and PAO1 *lasR*::Gm (b), viewed at 1,000× magnification. Fig. S5. Number of CFU of *P. aeruginosa* recovered after 24 hours of incubation in artificial sputum medium, with pig lung cube replaced with a corresponding volume of ASM.

  PDF, 1.6M
