# Supplementary material for: Development of an Ex Vivo Porcine Lung Model for Studying Growth, Virulence, and Signaling of Pseudomonas aeruginosa
Source: Infect Immun. 2014 Aug;82(8):3312–23. doi: 10.1128/IAI.01554-14 (PMC4136229; doi:10.1128/IAI.01554-14)
Supplement: Supplemental material [file IAI.01554-14_zii999090792so1.pdf]

**Development of an *ex vivo* porcine lung model for studying growth, virulence and signalling of *Pseudomonas aeruginosa***

Freya Harrison, Aneesha Muruli, Steven Higgins and Stephen P. Diggle

**Supplemental Material**

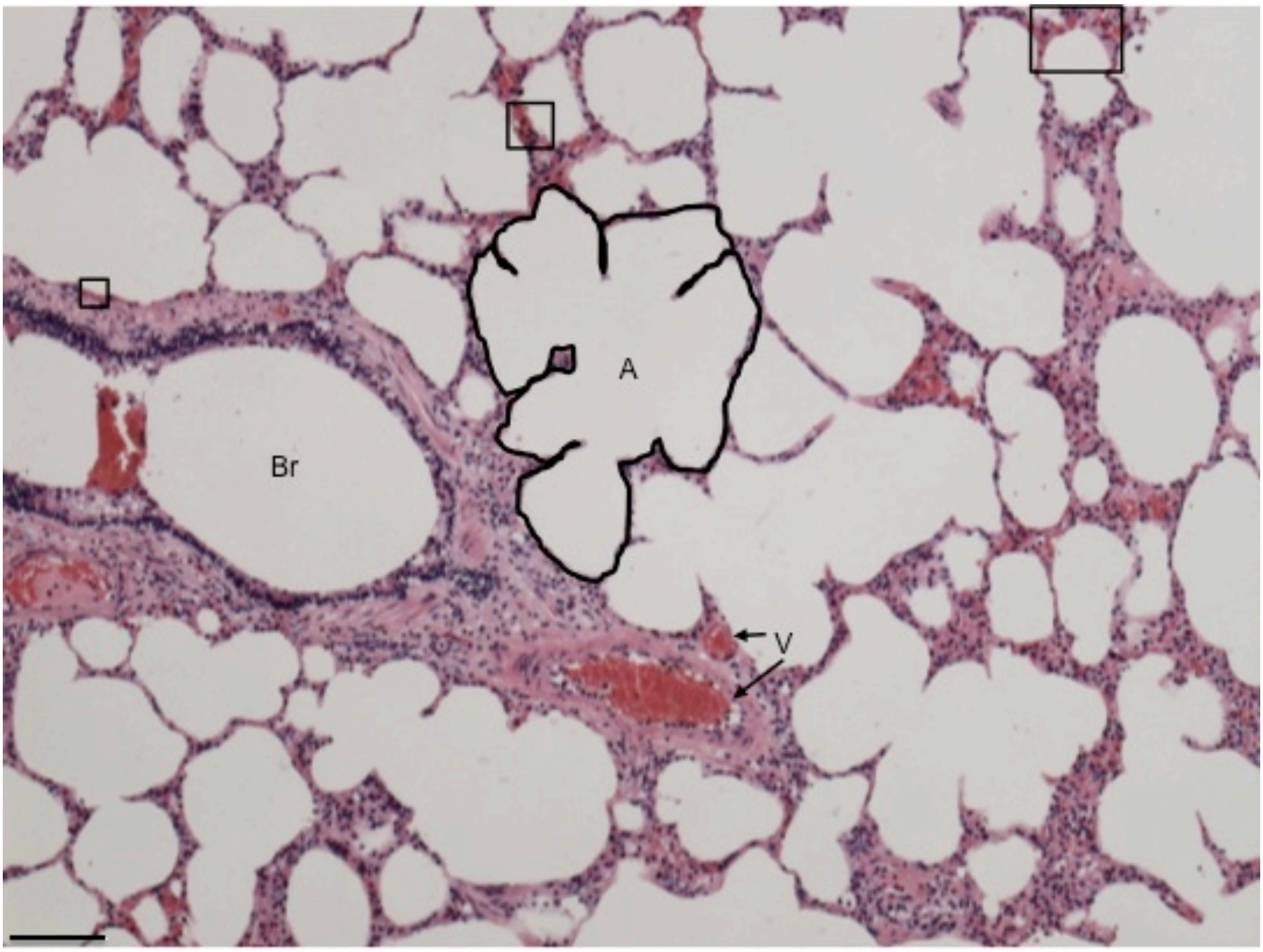


**Figure S1.** Micrograph of lung tissue prior to preparation. A section of tissue was snipped from the lung using scissors, immediately fixed in formalin and then sectioned and stained with H&E; this colors nuclei dark blue and other structures (cytoplasm, collagen etc.) pink. Tissue shown at 100X magnification with a 100$\mu$M scale bar. Note bronchiole (Br), examples of blood vessels (V) and lace-like pattern of alveoli defined by thin epithelium (example outlined, A). Some red blood cells (examples boxed) have been released from blood vessels by cutting the tissue. (Photo courtesy of V. Sovani).

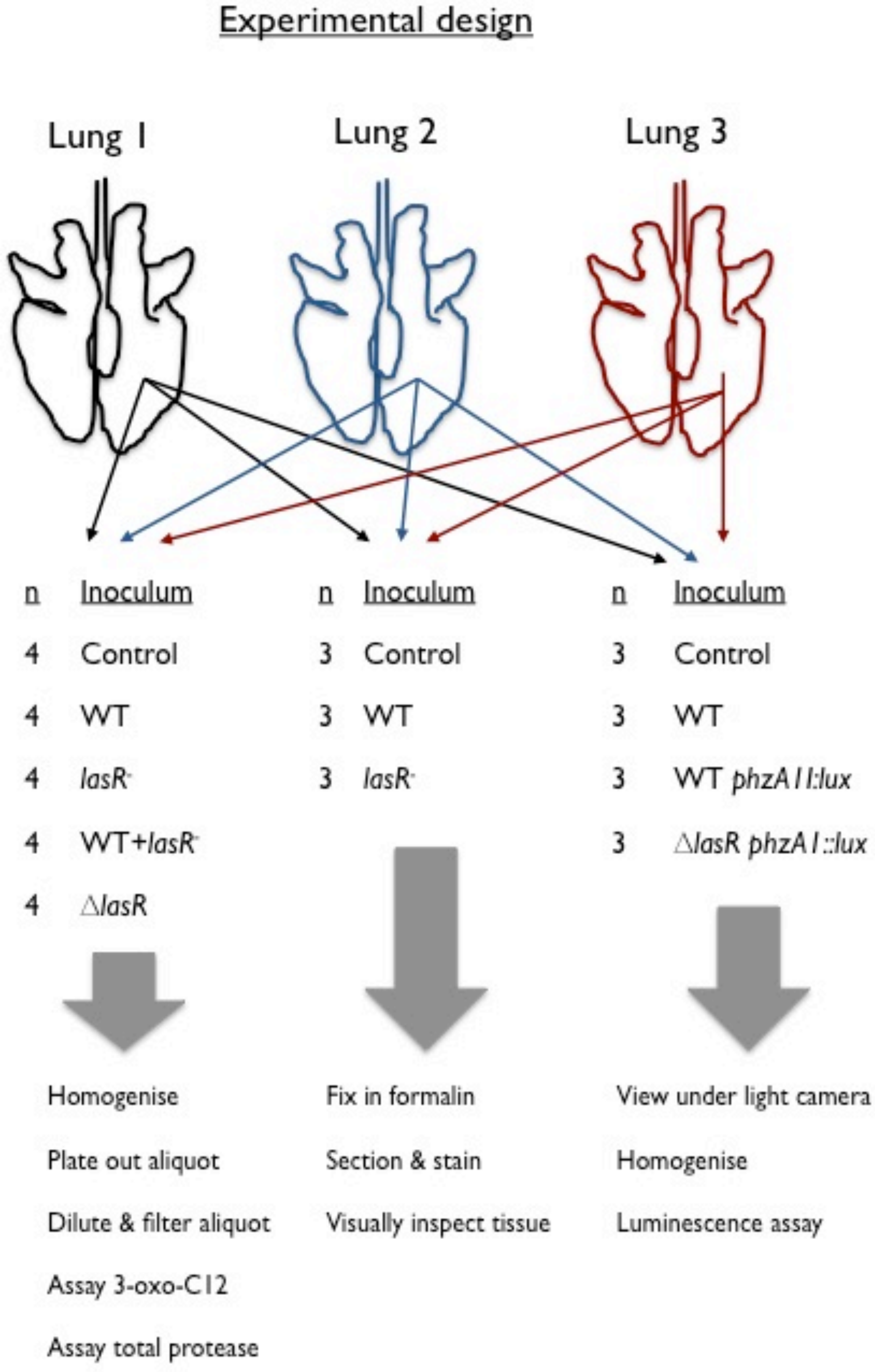


**Figure S2.** Schematic of the experiment, showing numbers of cubes dissected from lungs and assigned to different treatments/assays.

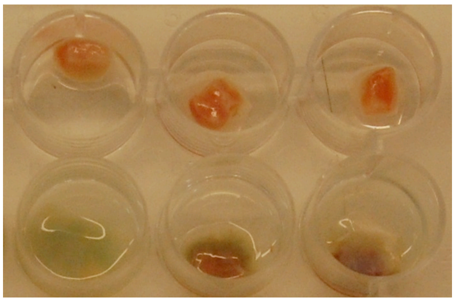

**Figure S3.** Example of lung cubes after 24 hours incubation at 37°C in ASM + washing in PBS. Top row shows mock-infected cubes, bottom row shows cubes infected with PAO1.

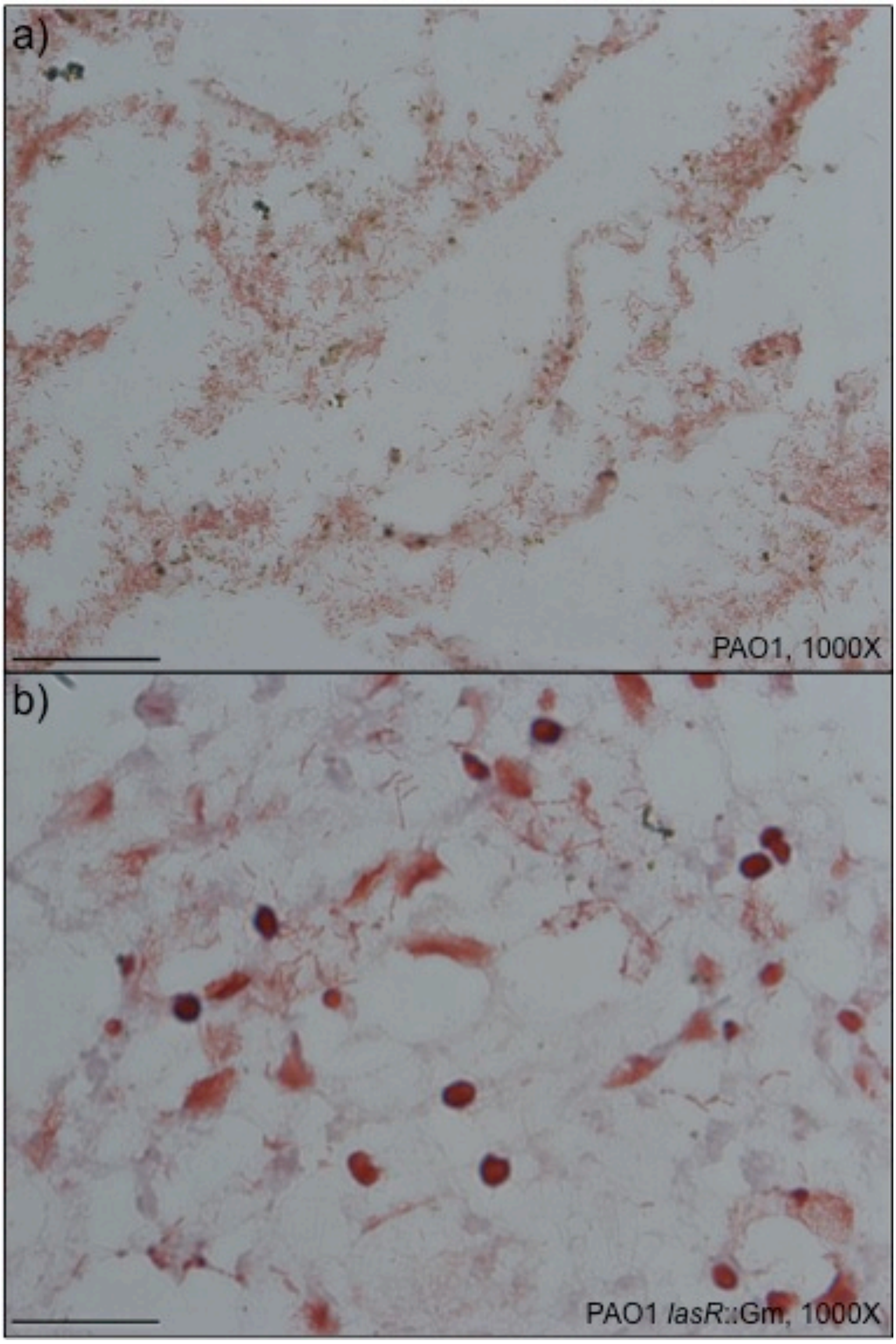


**Figure S4.** Gram-stained sections of tissue infected with a) WT PAO1 and b) PAO1 *lasR*::Gm, viewed at 1000X magnification (scale bar shows 20 $\mu$M). In both cases, large numbers of Gram-negative rods (pink) are visible.

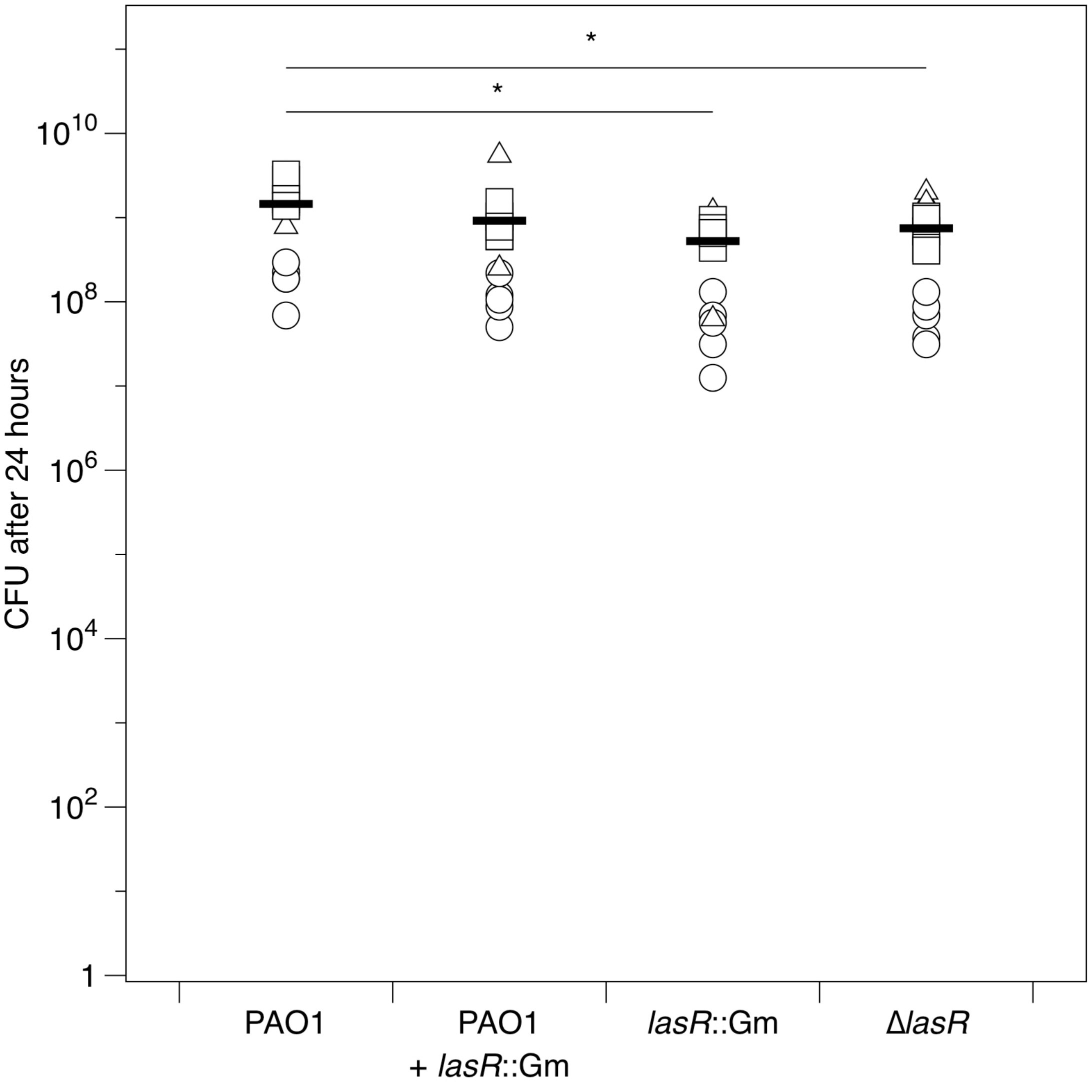


**Figure S5.** Number of colony-forming units (CFU) of *P. aeruginosa* recovered from after 24 hours of incubation in artificial sputum medium, with pig lung cube replaced with a corresponding volume of ASM. Different symbols show cubes from independent experimental replicates and bars denote overall means. Where pairwise differences between inocula were found to be significant ($p < 0.05$) using Tukey HSD tests, this is indicated with an asterisk.
